# Dual benefits of *Lysinibacillus xylanilyticus* strain GIC41 in mitigating Pythium root rot and enhancing plant growth across cultivation systems

**DOI:** 10.5511/plantbiotechnology.25.0316a

**Published:** 2025-09-25

**Authors:** Nusrat Ahsan, Stephany Angelia Tumewu, Ayaka Hieno, Masafumi Shimizu

**Affiliations:** 1 Center for Environmental and Societal Sustainability, Gifu University, Gifu 501-1193, Japan; 2 Faculty of Applied Biological Sciences, Gifu University, Gifu 501-1193, Japan

**Keywords:** biocontrol, hydroponic, *Lysinibacillus xylanilyticus*, plant growth-promoting bacteria, Pythium root rot

## Abstract

The *Lysinibacillus xylanilyticus* strain GIC41 has been previously reported to promote spinach growth. This study evaluated GIC41’s potential as a biostimulant by assessing its ability to mitigate Pythium root rot and enhance plant growth across various cultivation systems. In a pot experiment, GIC41 application to potting soil reduced the disease severity index (DSI) by approximately 52% in spinach seedlings 15 days post-pathogen inoculation (dpi). Similarly, introducing GIC41 into hydroponic nutrient solutions decreased the DSI in tomato seedlings from 61% to 15% at 14 dpi. Reisolation experiments and quantitative real-time PCR analysis confirmed that GIC41 significantly suppressed root colonization by *Pythium aphanidermatum* in both spinach and tomato seedlings. Microscopic analysis showed that GIC41 treatment inhibited pathogen mycelial colonization and caused morphological abnormalities in about 93% of encysted zoospores on the tomato rhizoplane. Although GIC41 exhibited no direct anti-oomycete activity in dual culture, it produced protease. Notably, GIC41 treatment significantly improved plant growth, increasing tomato shoot dry weight and stem diameter by 47% and 43%, respectively. These findings suggest that GIC41 is a promising biostimulant, offering dual benefits of disease mitigation and growth promotion across different crops and cultivation systems.

## Introduction

Root rot caused by *Pythium* species is among the most destructive plant diseases, leading to significant losses in global agricultural production (Wu et al. 2020). This pathogen infects a wide range of plant species under diverse environmental conditions and is particularly devastating for crops grown in greenhouses and nurseries (Parveen and Sharma 2015; Rai et al. 2020). Although control measures such as synthetic fungicides, crop rotation, and soil solarization have been employed, these strategies face limitations, including the development of fungicide resistance, the pathogen’s broad host range, and potential harm to beneficial soil microorganisms (Katan 2000; Matthiesen et al. 2016; Ma and Michailides 2005).

In recent years, plant growth-promoting bacteria (PGPB) have gained attention as sustainable agricultural solutions due to their dual role in enhancing plant growth and suppressing diseases (Backer et al. 2018). PGPB promotes plant growth through various mechanisms, including phytohormone production, nutrient solubilization, and nitrogen fixation (Olanrewaju et al. 2017). Moreover, studies have shown that PGPB can effectively suppress plant diseases through niche competition, antibiosis, pathogen cell wall degradation, and stimulation of plant immunity (Köhl et al. 2019). The use of PGPB with multiple beneficial traits is particularly promising, as it simultaneously addresses disease control and crop productivity challenges.

Endospore-forming bacteria, such as *Bacillus* and *Paenibacillus* species, are of particular interest as PGPB due to their resilience under environmental stress and suitability for commercial formulation (Etesami et al. 2023; Khan et al. 2020; Li et al. 2020). The genus *Lysinibacillus* is another group of endospore-forming bacteria that researchers have recently explored as microbial inoculants for crop production (Ahsan and Shimizu 2021; Pantoja-Guerra et al. 2023). In a previous study, we isolated the endospore-forming strain GIC41 from the genus *Lysinibacillus*, identified as *Lysinibacillus xylanilyticus* through 16S rRNA sequence analysis. This strain exhibited notable plant growth-promoting effects in spinach, particularly by enhancing root development (Ahsan et al. 2021). Considering the importance of robust root systems for plant growth and disease resistance, we hypothesized that GIC41 might also protect plants against root diseases.

The present study aimed to comprehensively evaluate the potential of *L. xylanilyticus* GIC41 as a biostimulant by investigating three key aspects: (1) its ability to reduce damage caused by the root rot pathogen *Pythium aphanidermatum* across different cultivation systems, (2) its growth-promoting effects on multiple crops, and (3) the mechanisms underlying its disease-mitigating activity. To demonstrate the broad applicability of this strain, experiments were conducted using both soil-based and hydroponic systems with spinach and tomato as model crops.

## Materials and methods

### Bacterial strain, culture conditions, and inoculum preparation

*L. xylanilyticus* strain GIC41 was cultured in nutrient broth (Nissui Pharmaceutical, Tokyo, Japan) at 30°C for 48 h with continuous shaking at 200 rpm. Following incubation, the bacterial cells were harvested by centrifugation at 9,900×g for 10 min and washed twice with 10 mM MgCl_2_·6H_2_O to remove residual media components. The washed cells were then resuspended in the same 10 mM MgCl_2_·6H_2_O solution and adjusted to an optical density at 600 nm (OD_600_) of 0.5, corresponding to approximately 10^7^ colony forming units (CFU) ml^−1^. This suspension was subsequently used as the inoculum for biocontrol experiments. For the assessment of protease and cellulase activities, GIC41 was cultured in tryptic soy broth at 30°C for 24 h with shaking at 200 rpm. Post-incubation, the cells were collected via centrifugation and resuspended in sterile distilled water (SDW) to an OD_600_ of 0.5 for enzyme activity assays.

Additionally, *Mitsuaria* sp. strain TWR114, a known biocontrol bacterium (Marian et al. 2018), was included in this study for comparative analysis of cellulase and protease activities. The inoculum for TWR114 was prepared following the same protocol used for GIC41.

### Pathogen, culture conditions, and inoculum preparation

For pathogen challenge assays, the *P. aphanidermatum* isolate GUEh20To1, part of Hieno’s laboratory *Pythium* collection, was used. Isolate was cultured on corn meal agar (CMA) medium (composed of 20 g corn, 20 g agar, and 1 l distilled water) and incubated at 25°C in the dark for 24 h. Actively growing hyphal tips were excised using an 8-mm diameter cork borer. To induce zoospore production, ten mycelial discs were placed in a 9-cm glass Petri dish containing 30 ml of autoclaved pond water (a 2 : 1 mixture of distilled and filtered pond water) and incubated at 25°C in the dark. After 24 h, the zoospores were harvested, and their concentration was standardized to 10^4^ zoospores ml^−1^ for use in subsequent experiments.

### Efficacy of GIC41 against Pythium root rot in spinach

The mitigating effect of *L. xylanilyticus* strain GIC41 against *Pythium* root rot in spinach seedlings was assessed through the following procedure. Spinach seeds (*Spinacia oleracea* L. cv. Banchu-summer-sky) were surface sterilized by immersion in 70% (v/v) ethanol for 1 min, followed by treatment with 2% sodium hypochlorite for 5 min. The seeds were then thoroughly rinsed with SDW to remove any residual disinfectant. Subsequently, the seeds were placed on moist filter paper in Petri dishes and vernalized at 4°C for 24 h. After vernalization, three seeds were sown in each plastic pot (8 cm×7.5 cm) containing 150 g of double-autoclaved commercial soil (Saika Ichiban, Ibigawa Kogyou Co., Ltd., Gifu, Japan). The pots were maintained in a controlled-environment chamber (Biotron LH-220S, Nippon Medical and Chemical Instruments Co., Ltd., Osaka, Japan) set at 23°C under a 12-h light/12-h dark cycle. Seven days post-sowing, each pot was drenched with 5 ml of *P. aphanidermatum* zoospore suspension, followed by 5 ml of the GIC41 bacterial suspension. For the inoculated control, pots received the zoospore solution and were treated with 5 ml of 10 mM MgCl_2_·6H_2_O solution instead of the GIC41 suspension. Uninoculated control pots were drenched with SDW and 10 mM MgCl_2_·6H_2_O solution. All pots were placed in the same growth chamber and watered regularly throughout the experiment. Disease symptoms were monitored daily through visual assessment for up to 15 days post-inoculation (dpi) using a disease severity scale ranging from 0 to 4, where 0=no disease symptoms (healthy), 1=slightly stunted growth compared to healthy seedlings, 2=moderate stunting or chlorosis, 3=severe stunting or wilting, and 4=completely wilted or dead (Supplementary Figure S1). The disease severity index (DSI) was calculated using the following formula: DSI=[Σ (the number of diseased plants in each disease scale × disease scale)/(total number of plants investigated × the highest disease scale)]×100. Three pots were used as replicates for each treatment, and the experiment was repeated three times. Differences in DSI between the inoculated control and GIC41-treated groups were analyzed using Student’s *t*-test (*p*<0.05).

### Effect of GIC41 on the root colonization of *P. aphanidermatum* in spinach

The extent of root colonization by *Pythium* in GIC41-treated spinach seedlings was evaluated and compared with that in inoculated control seedlings. Spinach seedlings were inoculated with both the pathogen and GIC41 following the previously described protocol. At 3 to 4 dpi, the seedlings were carefully uprooted, and their roots were gently rinsed with running tap water to remove adhering soil particles. After cleaning, lateral roots were trimmed, and the primary root was cut into 1-cm segments. For each seedling, five root segments were selected: two from the upper portion, two from the lower portion, and one from the middle section of the primary root. These segments were placed on a *Pythium* selective medium consisting of CMA supplemented with nystatin (10 mg l^−1^) and miconazole (1 mg l^−1^) (Senda et al. 2009). The plates were incubated in the dark at 23°C for 24 h. Following incubation, hyphal growth from each root segment was examined under a microscope to confirm the presence of the pathogen. The reisolation frequency was calculated as the percentage of root segments from which *Pythium* was successfully recovered. Five plants were analyzed per treatment, and the experiment was repeated three times to ensure reproducibility. Statistical differences in the isolation frequency between the inoculated control and GIC41-treated groups were assessed using Student’s *t*-test (*p*<0.05).

### Efficacy of GIC41 against Pythium root rot in tomato

The suppressive effect of *L. xylanilyticus* strain GIC41 against *Pythium* root rot in hydroponically grown tomato seedlings was evaluated through a controlled experimental setup. Tomato seeds (*Solanum lycopersicum* L. cv. Reigetsu) were surface sterilized with 10% H_2_O_2_ for 20 min and thoroughly rinsed three times with SDW. The sterilized seeds were placed on moist filter paper in Petri dishes and incubated in the dark at 25°C for two days to promote germination. Seven germinated seeds were then transferred onto urethane sponges (25 mm cubes with H-shaped slits; LFS-023-4, Living-farm Co., Ltd., Tokyo, Japan) positioned in hydroponic containers (LFS-013-2, Living-farm) filled with SDW. These containers were maintained in a controlled environment chamber (CLE-303; Tomy Seiko Co., Ltd., Tokyo, Japan) at 25°C under a 12-h light/12-h dark cycle, with SDW replenished every three days. After seven days of growth, the four most vigorous seedlings in each container were retained by removing the three weaker ones. Subsequently, the SDW in each container was replaced with 450 ml of a 150-fold diluted nutrient solution (LFS-106L; Living-farm), and 5 ml of the GIC41 bacterial suspension was added. For the untreated control, 5 ml of sterile 10 mM MgCl_2_·6H_2_O solution was added instead of the GIC41 suspension. All containers were maintained under the same conditions, with 3 ml of undiluted nutrient solution added weekly. Seven days after GIC41 treatment, each container was challenge-inoculated with 10 ml of *P. aphanidermatum* zoospore suspension. To maintain nutrient availability, 3 ml of undiluted nutrient solution was added every seven days. Additionally, untreated and GIC41-treated plants without pathogen inoculation were prepared as controls. At 14 dpi, disease severity, shoot dry weight, and stem diameter were measured. Disease severity was visually assessed on a scale from 0 to 3: 0 indicated no disease symptoms (healthy), 1 indicated slightly stunted growth compared to healthy seedlings, 2 indicated severely stunted growth, and 3 indicated completely wilted or dead plants. The DSI was calculated using the same formula as in the spinach pot experiment. Each treatment included two containers (eight seedlings total) as replicates, and the experiment was repeated three times for accuracy. Statistical differences in DSI between the inoculated control and GIC41 treated groups were analyzed using Student’s *t*-test (*p*<0.01), while variations in shoot dry weight and stem diameter among the four treatments were assessed using Tukey’s test (*p*<0.05).

### Effect of GIC41 treatment on root colonization by *P. aphanidermatum* mycelia in tomato seedlings

The effect of GIC41 treatment on root colonization by pathogen mycelia in tomato seedlings was evaluated through microscopic observation. Tomato seedlings were grown hydroponically and inoculated with the pathogen and GIC41 as previously described. At 48 and 72 h post-inoculation (hpi), root tips were cut into 1-cm segments, mounted on glass slides, and examined under a microscope (U-TV1X-2; Olympus Corporation, Tokyo, Japan). The extent of colonization in each root segment was scored on a scale of 0 to 3, where 0 indicated no mycelia on the root segment, 1 indicated less than 50% of the root segment was covered by mycelia, 2 indicated 50% to less than 75% coverage, and 3 indicated 75% to 100% coverage. The root colonization index (RCI) was calculated using the formula: RCI=[Σ (the number of segments in each scale × colonization scale/ (total number of root segments investigated × the highest colonization scale)]×100. Ten root segments were examined per seedling. Four plants were used for each treatment, and the experiment was repeated three times. Differences in RCI between the inoculated control and GIC41 treatment groups were analyzed using Student’s *t*-test (*p*<0.01).

### Effect of GIC41 on root colonization by *P. aphanidermatum* in tomato seedlings

The pathogen population in the roots of GIC41-treated and untreated tomato seedlings was compared using probe-based quantitative real-time PCR (qRT-PCR). Tomato seedlings were grown hydroponically and inoculated with the pathogen and GIC41 as previously described. Roots were collected from each seedling at 24 and 48 hpi immediately frozen in liquid nitrogen, and stored at −80°C until DNA extraction.

DNA was extracted from the root samples using a bead-beating grinder (FastPrep-24™ 5G; MP Biomedicals, USA) in combination with the FastDNA® Green SPIN Kit for plant and animal tissue (MP Biochemicals), following the manufacturer’s instructions with slight modifications. If the samples were not sufficiently homogenized, the grinding time was extended by 5 min to ensure complete comminution.

The primer and probe sets used for the amplification of *P. aphanidermatum* and tomato DNA are listed in Supplementary Table S1. The reaction mixture had a final volume of 20 µl and contained 10 µl of Probe qPCR Mix (TaKaRa Bio Inc., Kusatsu, Japan), 0.4 µl of forward primer (10 µM), 0.4 µl of reverse primer (10 µM), 1.6 µl of TaqMan probe (5 µM), 0.4 µl of ROX reference dye, 2 µl of DNA template, and 5.2 µl of SDW. qRT-PCR was performed using the StepOnePlus™ Real-Time PCR system (Applied Biosystems). The cycling conditions consisted of an initial denaturation at 95°C for 30 s, followed by 40 cycles of denaturation at 95°C for 5 s and annealing/extension at 62°C for 30 s. For each treatment, roots collected from four seedlings were pooled and used as a sample for DNA extraction, and the qRT-PCR reaction was performed with three technical replicates. The entire experiment was conducted three times independently, with each repetition considered a biological replicate.

The cycle threshold (Ct) values were automatically calculated by the StepOnePlus™ software and exported to Microsoft Excel (Office 365) for further analysis. Standard curves were created by plotting the Ct values of serially diluted DNA (ranging from 200 ng to 0.002 ng) of *P. aphanidermatum* or tomato roots. The regression line generated from the standard curve was used to calculate the amount of target DNA in each sample based on its Ct value. The pathogen load in the roots was expressed as picograms (pg) of *P. aphanidermatum* DNA per 100 ng of tomato DNA.

### Effect of GIC41 treatment on the cystospores of *P. aphanidermatum*

The effect of GIC41 on the morphology of the pathogen’s cystospores (encysted zoospores) was also assessed microscopically at 24 hpi. Tomato seedlings were grown hydroponically and inoculated with the pathogen and GIC41 as previously described. For each seedling, 50 cystospores attached to the roots were randomly selected, and the number of cystospores displaying abnormal morphology (e.g., shrunken or irregular shapes) was recorded. Images of *Pythium* encysted zoospores in various root regions were captured using a microscope (U-TV1X-2; Olympus) equipped with a DP2-SAL camera (Olympus). Four plants were analyzed per treatment, and the experiment was repeated three times. Differences in the percentage of cystospores with abnormal morphology between the inoculated control and the GIC41-treated samples were evaluated using Student’s *t*-test (*p*<0.01).

### Dual culture assay

The inhibitory activity of GIC41 against the pathogen was evaluated using a dual culture assay on agar medium composed of a 1 : 1 (v/v) mixture of nutrient agar (NA) and potato dextrose agar (PDA). GIC41 was pre-cultured on NA for 24 h at 30°C, while the pathogen was pre-cultured on PDA for 24 h at 25°C in the dark. Mycelial plugs, 8 mm in diameter, were cut from the leading edge of the pathogen colony using a cork borer and placed on one side of the aforementioned mixed medium of NA and PDA (referred to as NA·PDA) in a 9-cm Petri dish. A loopful of GIC41 cells from the NA colony was streaked on the opposite side of the NA·PDA medium. The plates were then incubated at 23°C for five days. Plates without the bacterial strain served as controls to assess pathogen growth in the absence of GIC41.

### Protease and cellulase activity assays

The protease and cellulase activities of GIC41 were also evaluated, with *Mitsuaria* sp. TWR114 used as a positive control. Protease activity was assessed using a plate assay with skimmed milk agar (SMA) following the method of Morris et al. (2012) with slight modifications. Cellulase activity was tested using a plate assay with carboxymethylcellulose agar (CMCA), based on the procedures described by Izquierdo-García and Moreno-Velandia (2024), also with minor modifications. For both assays, 10 ml of SMA or CMCA were poured into 6-cm Petri dishes and allowed to solidify in a laminar flow cabinet. A 5-mm hole was created in the center of each agar plate using a cork borer, and 30 µl of bacterial cell suspension OD_600_=0.5) was added into the hole. The plates were incubated at 30°C for three days. Protease activity was indicated by a clear zone forming around the inoculation site, while cellulase activity was evidenced by a yellow halo after staining with 0.5% (w/v) Congo red for 20 min, followed by washing with 1 M NaCl.

### Statistical analysis

All statistical analyses were conducted using EZR version 1.55 (Saitama Medical Center), a graphical user interface for *R* (The R Foundation for Statistical Computing, version 2.7-1), available at http://www.jichi.ac.jp/saitama-sct/SaitamaHP.files/statmedEN.html.

## Results

### Mitigating effect of GIC41 against Pythium root rot in spinach

**Figure 1:**
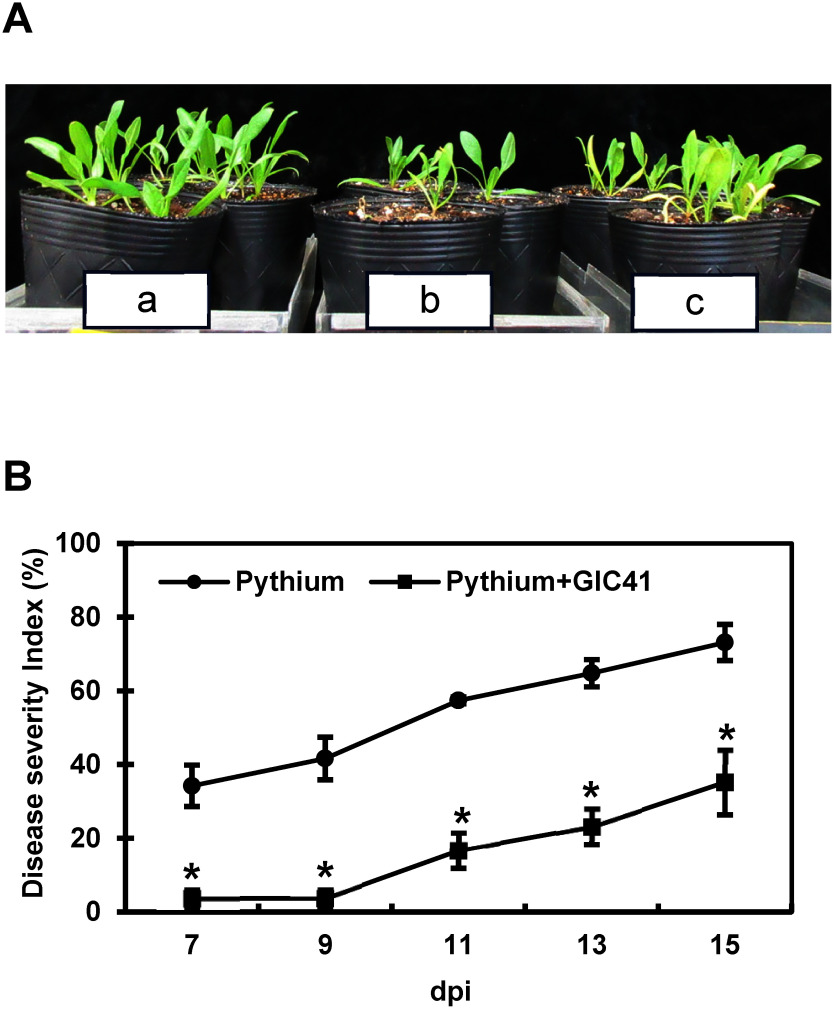
Figure 1. Mitigating effect of GIC41 treatment against Pythium root rot in spinach seedlings. (A) Overview of spinach seedlings (*Spinacia oleracea* L. cv. Banchu-summer-sky) at 15 dpi under different treatments; a, uninoculated control; b, inoculated control (Pythium only); c, GIC41-treated spinach seedlings. (B) Disease progression of inoculated control (Pythium only) and GIC41-treated spinach seedlings (Pythium+GIC41). Error bars represent the standard error of three independent experiments. An asterisk (*) indicates a significant difference between the control (Pythium) and GIC41 (Pythium+GIC41) treatments according to Student’s *t*-test (*p*<0.05).

In the inoculated control pots challenged with *P. aphanidermatum*, spinach seedlings exhibited severe stunted growth and chlorosis, with some plants wilting completely by 15 dpi (Figure 1A). The mean DSI in the inoculated control steadily increased from 34% at 7 dpi to 73% at 15 dpi (Figure 1B), indicating progressive disease development. In contrast, seedlings grown in GIC41-treated pots displayed noticeably healthier growth compared to those in the inoculated control (Figure 1A). Disease symptoms in the GIC41-treated seedlings developed much more slowly and remained significantly less severe throughout the cultivation period. By 15 dpi, the DSI in the GIC41 treatment group was reduced by approximately 52% compared to the inoculated control (Figure 1B). These findings strongly suggest that GIC41 has the potential to mitigate the development of *Pythium* root rot in spinach.

### Effect of GIC41 treatment on root colonization by *P. aphanidermatum* in spinach

**Figure 2:**
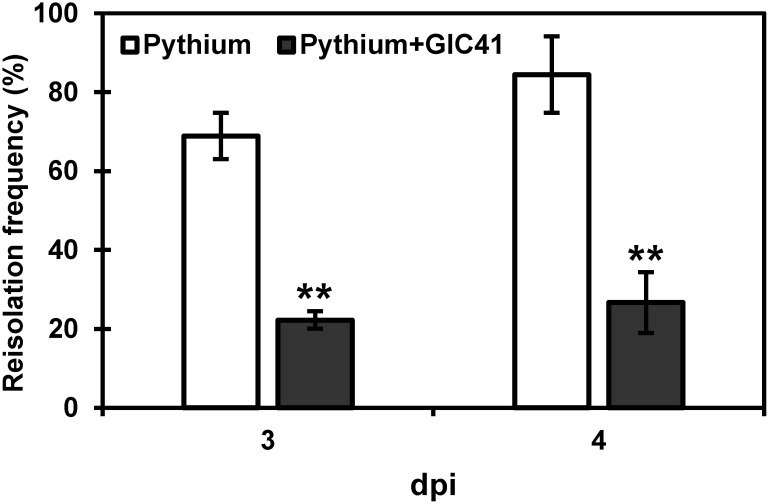
Figure 2. Effect of GIC41 treatment on root colonization of spinach by *Pythium aphanidermatum*. Error bars represent the standard error from three independent experiments. Asterisks (**) indicate significant differences between the control (Pythium) and GIC41 (Pythium+GIC41) treatments according to Student’s *t*-test (*p*<0.01).

To further assess the impact of GIC41 on root colonization by *P. aphanidermatum*, spinach seedlings were sampled at 3 and 4 dpi, and the pathogen was reisolated from their roots. At 3 dpi, the pathogen was detected in an average of 68% of the root segments from the inoculated control seedlings (Figure 2), which increased to 84% by 4 dpi. In contrast, the pathogen reisolation frequency from the root segments of GIC41-treated seedlings was significantly lower than that of the inoculated control seedlings at both 3 and 4 dpi (22% and 27% on average, respectively).

### Suppressive effect of GIC41 against Pythium root rot in tomato

**Figure 3:**
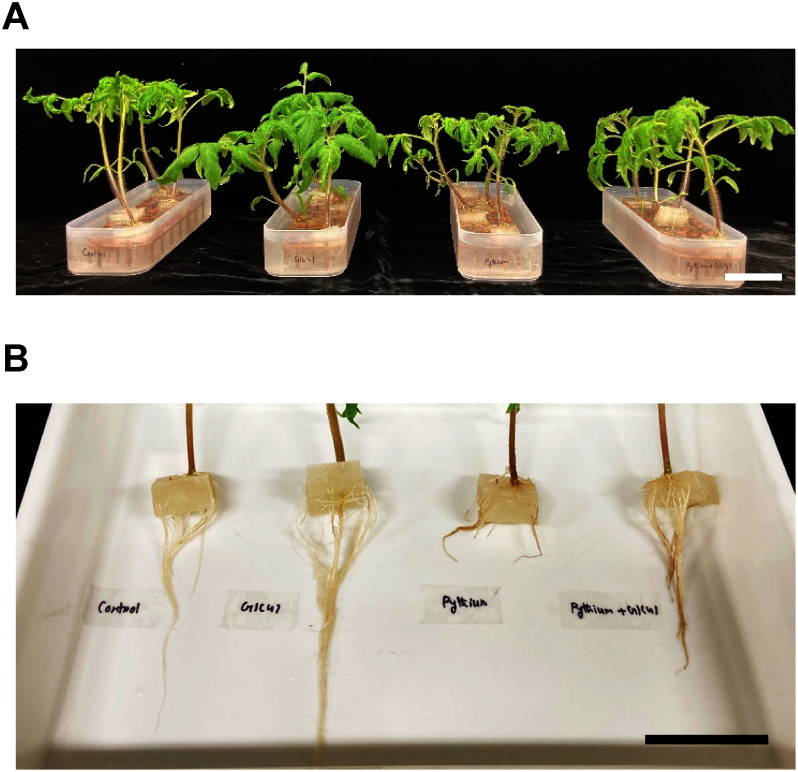
Figure 3. Effect of GIC41 treatment on shoot and root growth and root infection by *Pythium aphanidermatum* in hydroponically grown tomato seedlings. (A) Overview of tomato shoots under different treatments. (B) Overview of tomato roots under different treatments. In both panels, from left to right: uninoculated control seedlings, GIC41-treated seedlings, inoculated control seedlings (*Pythium* only), and GIC41-treated seedlings challenged with *P. aphanidermatum*. Scale bars: 5 cm.

Tomato seedlings in the inoculated control group exhibited stunted shoot growth (Figure 3A). Their shoot dry weight and stem diameter were reduced by approximately 47% and 43%, respectively, compared to those of seedlings in the uninoculated control (Table 1). Additionally, these seedlings showed severe root rot symptoms, leading to significant suppression of root development (Figure 3B, Table 1). The mean DSI for the inoculated control reached approximately 61% at 14 dpi (Table 1). In contrast, the shoot growth of seedlings in the *Pythium*+GIC41 treatment was markedly improved (Figure 3A, Table 1), and root rot symptoms were notably milder than in the inoculated control. The mean DSI in the GIC41-treated seedlings was significantly reduced to around 15% (Table 1). These results indicate that GIC41 effectively controls Pythium root rot in tomato plants.

**Table 1:** Table 1. Impact of GIC41 treatment on *Pythium* root rot severity, shoot dry weight, and stem diameter in hydroponically grown tomato seedlings.

Treatments	DSI	Shoot dry weight (mg)	Stem diameter (mm)
Uninoculated control	0	168.3±37.5 ab	3.6±0.3 b
GIC41	0	236.7±53.8 b	4.4±0.3 c
Pythium (inoculated control)	61.1±9.6	94.2±5.2 a	2.1±0.3 a
Pythium+GIC41	15.3±8.7**	181.3±41.3 ab	3.5±0.2 b

Each value represents the mean±standard error from three independent experiments. ** indicates a significant difference between the *Pythium* treatment and the *Pythium*+GIC41 treatment according to Student’s *t*-test (*p*<0.01). Different letters indicate significant differences among treatments (*p*<0.05, Tukey’s test). Data were collected at 14 dpi.

Furthermore, both shoot and root growth in GIC41-treated seedlings without pathogen inoculation were significantly enhanced compared to the uninoculated control (Figure 3, Table 1), suggesting that GIC41 also has a growth-promoting effect on tomato seedlings.

### Suppressive effect of GIC41 treatment against root colonization by *P. aphanidermatum* mycelia in tomato seedlings

In the inoculated control treatment, the root tips were heavily colonized by pathogen mycelia by 48 hpi, resulting in an average RCI of 55% (Supplementary Figure S2). By 72 hpi, pathogen colonization intensified, raising the average RCI to 71%. In contrast, the root tips of GIC41-treated seedlings showed considerably less mycelial coverage at both 48 and 72 hpi. The average RCI in the GIC41 treatment was significantly lower, measuring 25% at 48 hpi and 47% at 72 hpi.

### Reduction of the *P. aphanidermatum* population in tomato roots by GIC41 treatment

**Figure 4:**
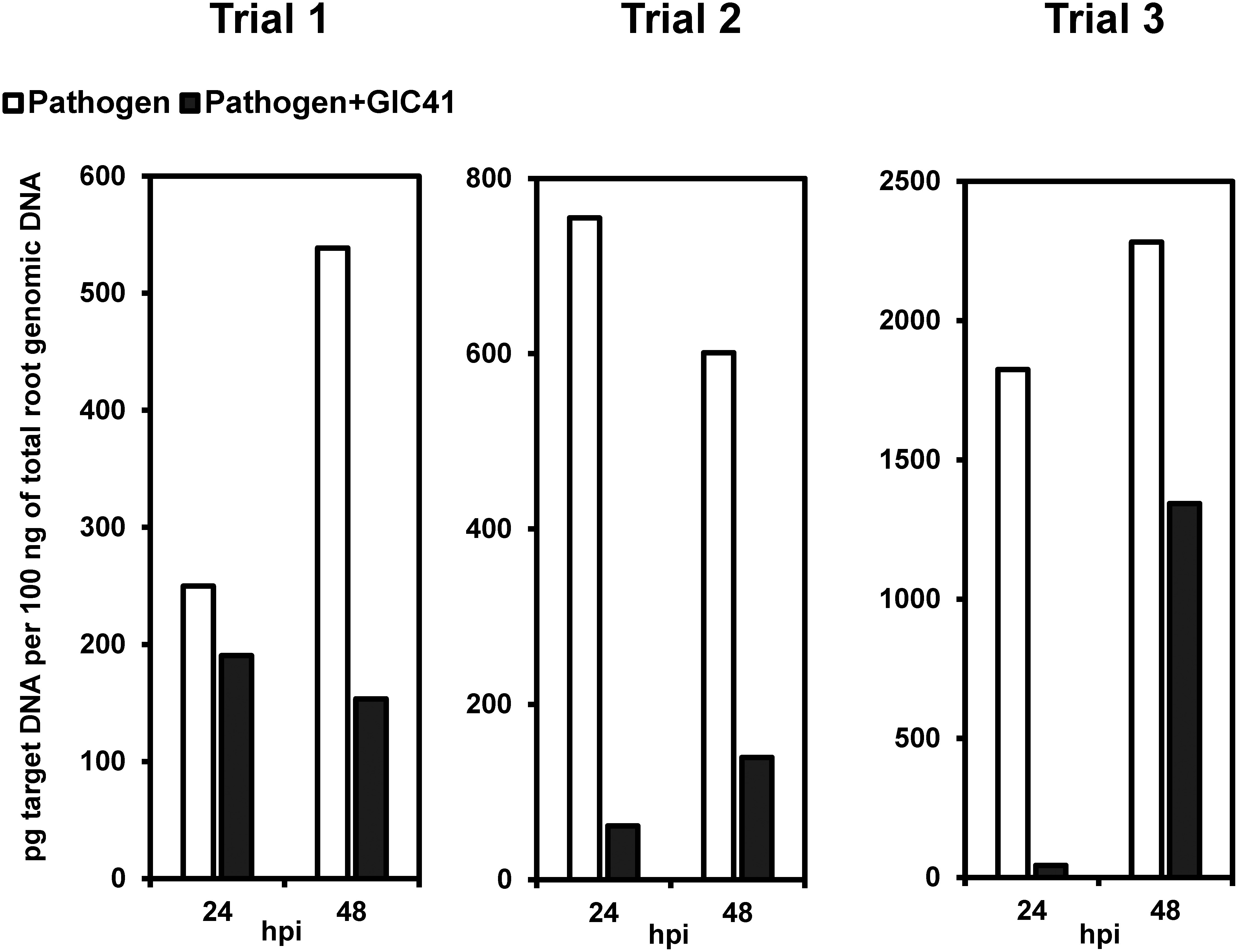
Figure 4. Reduction of *Pythium aphanidermatum* population in tomato seedling roots following GIC41 treatment. Quantitative assessment of the *P. aphanidermatum* population in the roots of hydroponically grown tomato seedlings, demonstrating significant suppression of *Pythium* colonization by GIC41 treatment.

The amounts of *P. aphanidermatum* target DNA relative to 100 ng of tomato root genomic DNA varied across replicate experiments (Figure 4). However, in all experiments, the amount of pathogen DNA in tomato roots was significantly higher in the inoculated control (*Pythium*) than in the GIC41-treated group (*Pythium*+GIC41) at both 24 and 48 hpi. This consistent reduction in pathogen DNA levels suggests that GIC41 treatment effectively suppressed root colonization by *P. aphanidermatum*.

### Effect of GIC41 treatment on the cystospores of *P. aphanidermatum*

**Figure 5:**
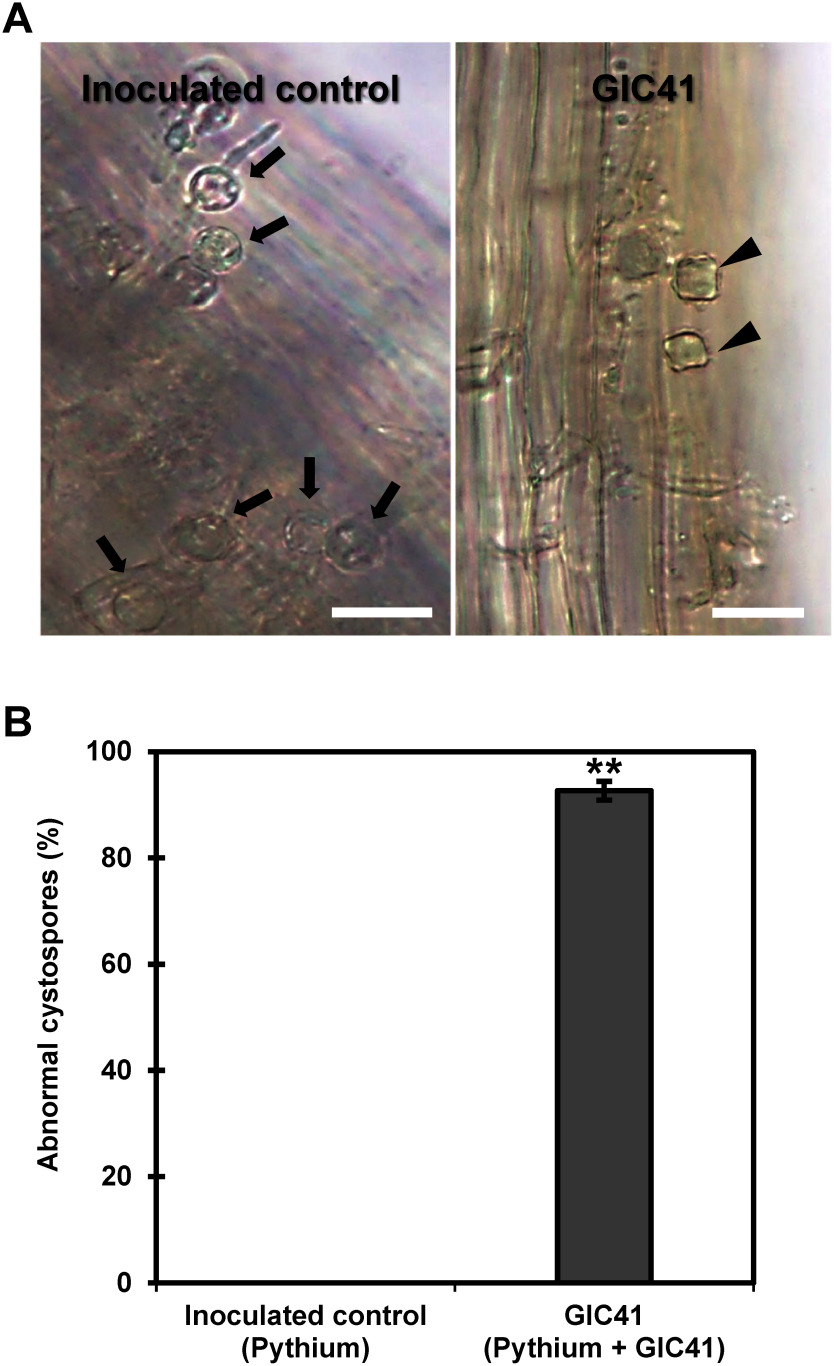
Figure 5. Impact of GIC41 on the morphology of *Pythium aphanidermatum* cystospores attached to tomato seedlings roots. (A) Microscopic images of cystospores on the roots of inoculated control seedlings (left) and GIC41-treated seedlings (right). Arrows indicate cystospores with normal round morphology, while arrowheads indicate abnormally shrunken cystospores. Scale bars=20 µm. (B) Percentage of abnormal cystospores observed at 24 hpi. Each value represents the mean±standard error from three independent experiments. ** indicates a significant difference between the inoculated control (Pythium) and GIC41 (Pythium+GIC41) treatments according to Student’s *t*-test (*p*<0.01).

Microscopic observation of *P. aphanidermatum* cystospores attached to tomato roots at 24 hpi revealed distinct differences between treatments. In the inoculated control, all observed cystospores maintained a normal round morphology (Figure 5A, B). In contrast, cystospores in the GIC41-treated roots appeared shrunken and irregular in shape. The incidence of these abnormal cystospores in the GIC41 treatment averaged approximately 93% (Figure 5B).

### Inhibitory activity of GIC41 against the hyphal growth of *P. aphanidermatum*

In the dual culture assay of GIC41 and *P. aphanidermatum* on NA·PDA medium, GIC41 exhibited no inhibitory activity against the hyphal growth of the pathogen (Supplementary Figure S3).

### Cellulase and protease activities of GIC41

**Figure 6:**
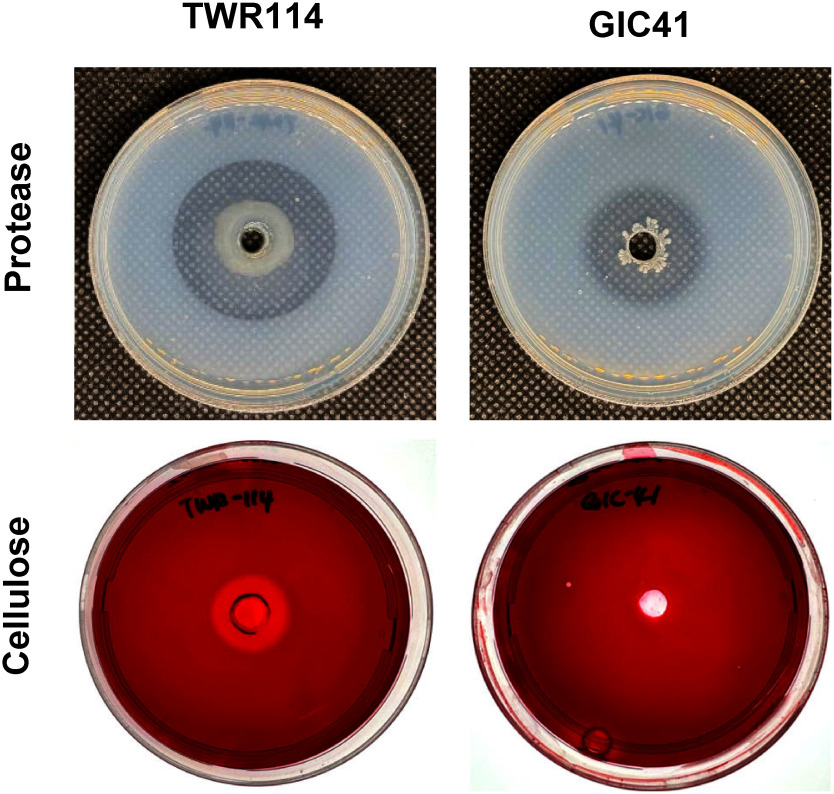
Figure 6. Protease and cellulase activities of GIC41. Top: Protease activity on skim milk agar medium. Bottom: Cellulase activity on carboxymethyl cellulose agar. *Mitsuaria* sp. TWR114 was used as a positive control.

As a positive control, *Mistuaria* sp. TWR114 produced a yellow halo on CMCA and a clear zone on SMA (Figure 6), indicating the production of both cellulase and protease. In contrast, GIC41 produced a clear zone on SMA but did not form a yellow halo on CMCA, suggesting that GIC41 produces protease but lacks cellulase activity.

## Discussion

Previously we reported that *L. xylanilyticus* strain GIC41, isolated from paddy field soil, exhibited a growth-promoting effect on spinach (Ahsan et al. 2021). In this study, we evaluated the biocontrol potential of GIC41 against Pythium root rot caused by *P. aphanidermatum*. Our results demonstrate that the incorporation of GIC41 into soil and hydroponic nutrient solutions effectively mitigate the development of Pythium root rot in spinach and tomato seedlings, respectively. Several *Lysinibacillus* species, such as *L. sphaericus* and *L. fusiformis*, have been reported to possess biocontrol activity against various fungal and bacterial diseases (Naureen et al. 2017; Passera et al. 2021; Shabanamol et al. 2018; Singh et al. 2013). Among these, *L. sphaericus* is the most widely studied biocontrol agent. For instance, Shabanamol et al. (2017) demonstrated that the application of *L. sphaericus* significantly suppressed rice sheath blight caused by *Rhizoctonia solani*. Similarly, Sakthivel et al. (2018) reported that two strains of *L. sphaericus*, isolated from sea sand and the chili rhizosphere in India, exhibited notable biocontrol effects against bacterial wilt of chili caused by *Ralstonia solanacearum* (Sakthivel et al. 2018). More recently, Moreno-Velandia et al. (2024) highlighted the biocontrol potential of a bacterial consortia, including *Bacillus velezensis* strain Bs006, *Pseudomonas fluorescens* strain Ps006, and *L. xylanilyticus* strain Br042, against clubroot in broccoli caused by *Plasmodiophora brassicae* (Moreno-Velandia et al. 2024). However, to the best of our knowledge, no prior studies have reported the biocontrol effect of a single strain of *L. xylanilyticus* against plant diseases. Therefore, this study is the first to demonstrate the mitigating effect of *L. xylanilyticus* strain GIC41 on diseases caused by phytopathogenic *Pythium* spp.

In this study, to elucidate how GIC41 mitigates Pythium root rot, we investigated its effect on root infection by *P. aphanidermatum* through pathogen reisolation experiments and qRT-PCR analysis. Our results revealed that root colonization by the pathogen was significantly reduced in both spinach and tomato seedlings treated with GIC41. Microscopic observations further demonstrated that mycelial growth on the roots of GIC41-treated tomato seedlings was notably disrupted. Additionally, most of the pathogen’s zoospores (cystospores) attached to these roots appeared shrunken and irregular in shape. Although the infection process on spinach roots was not examined microscopically, it is likely that the impact of GIC41 treatment on cystospore morphology and mycelial colonization contributed to the mitigation of Pythium root rot in both spinach and tomato seedlings. In this context, our results suggest that ensuring root colonization of GIC41 by applying the strain to seeds or transplants prior to planting, which allows for efficient interruption of the early stages of pathogen infection, may be important to maximize the protective effect.

The mechanisms by which antagonistic bacteria control plant diseases have been extensively studied and include the production of antibiotics, lytic enzymes, hydrogen cyanide, niche competition, and/or the stimulation of host plant immunity (Bonaterra et al. 2022; Compant et al. 2005; Roca-Couso et al. 2021). While niche competition may be one of the possible mechanisms by which GIC41 reduced root colonization by the pathogen, additional factors are likely involved. As mentioned above, most of the pathogen’s cystospores attached to tomato roots showed abnormal morphology. This fact suggests that beyond competition for nutrients and space, other inhibitory mechanisms may be affecting pathogen viability and development.

Species of *Lysinibacillus* are known to produce a range of antimicrobial metabolites, such as surfactin, pyrrolo[1,2-a] pyrazine-1,4-dione, digitoxin, and terbinafine (Akintayo et al. 2022; Che et al. 2017; El-Sayed et al. 2022; Mahmoud et al. 2023; Mechri et al. 2017), as well as lytic enzymes like chitinase, cellulase, and protease (Biswas et al. 2024; Mechri et al. 2017; Phazna et al. 2022; Shabanamol et al. 2017). Based on this information, we hypothesized that GIC41 might directly damage *P. aphanidermatum* mycelia and cystospores through the production of antimicrobial metabolites and lytic enzymes. However, interestingly, the dual culture assay indicated that GIC41 does not produce diffusible antimicrobial metabolites that directly inhibit hyphal growth of the pathogen. Instead, the enzyme activity assay revealed that GIC41 produces protease. This protease activity may target structural proteins in the cell walls of the pathogen and contribute to the observed morphological abnormalities in the cystospores and the reduced hyphal growth on the rhizoplane, although further investigation is needed to confirm this relationship.

The secretion of antimicrobial compounds from host plant roots potentially induced by GIC41 may represent another potential mechanism contributing to the suppression of pathogen colonization. Previous studies have shown that inoculation with certain antagonistic microbial strains or arbuscular mycorrhizal fungi can enhance the secretion of antimicrobial compounds from host plant roots, thereby inhibiting pathogen progression and suppressing soil-borne diseases (Ling et al. 2011; Windisch et al. 2017; Zhang et al. 2012). As its name suggests, *L. xylanilyticus* is well known for secreting the xylan-degrading enzyme xylanase (Lee et al. 2010). Notably, xylanase produced by certain pathogenic and non-pathogenic microbes has recently been reported to stimulate plant defense responses by functioning as a microbe-associated molecular pattern (MAMP) elicitor (Guo et al. 2021; Zhao et al. 2024). This raises the possibility that xylanase secreted by GIC41 could similarly act as a MAMP elicitor, triggering plant defense mechanisms and inducing the secretion of anti-oomycete metabolites from roots. Further research is needed to clarify the detailed mode of action of GIC41 in this context.

If our hypothesis about the involvement of protease and the likely produced xylanase in the biocontrol mechanism of GIC41 is correct, the culture supernatant containing secreted enzymes could be used as an alternative to direct bacterial application. However, this approach may not be realistic because the applied enzymes would easily lose their activity due to rapid non-biological and biological denaturation as well as degradation by proteolytic microorganisms residing in the environment (Olagoke et al. 2020), thus requiring regular application to maintain the disease protective effect. In this regard, the use of live GIC41 cells would be more practical for long-term protection, as the strain can continuously secrete these enzymes while surviving on the roots.

In addition to mitigating Pythium root rot, GIC41 also demonstrated significant growth-promoting effects on tomato seedlings. Tomato plants treated with GIC41 exhibited a notable increase in both shoot and root growth. This finding aligns with previous reports where soil application of GIC41 enhanced shoot and root biomass in spinach (Ahsan et al. 2021). These results suggest that GIC41 can directly and indirectly promote growth across various crop species, highlighting its potential as a biostimulant to improve crop productivity. However, the specific mechanism underlying GIC41’s plant growth-promoting effect remains unclear. Recent studies have reported that xylanase produced by *Trichoderma asperellum* promotes lateral root growth in poplar (Guo et al. 2021). It is plausible that the xylanase secreted by GIC41 may exhibit a similar function, contributing to both disease suppression and plant growth promotion. This dual functionality makes GIC41 an intriguing candidate for future mechanistic studies aimed at understanding its role in plant health and development.

In conclusion, this study demonstrates that *L. xylanilyticus* strain GIC41 is a promising biocontrol agent that effectively mitigates Pythium root rot in both spinach and tomato, while simultaneously promoting plant growth. The dual beneficial effect of disease control and growth promotion highlights GIC41’s potential as a valuable tool for sustainable agriculture. Future research should focus on uncovering the molecular mechanisms underlying GIC41’s biocontrol and growth-promoting effects, particularly the potential dual role of xylanase, which will further enhance our understanding of GIC41’s mode of action and optimize its application in agricultural settings.

## Abbreviations

CMA: corn meal agar
CMCA: carboxymethylcellulose agar
DSI: disease severity index
MAMP: microbe-associated molecular pattern
NA: nutrient agar
PDA: potato dextrose agar
PGPB: plant growth-promoting bacteria
RCI: root colonization index
SMA: skimmed milk agar

## Description of supplementary files

Supplementary Table S1. Primer and probe sets used for DNA amplification of *Pythium aphanidermatum* and tomato.

Supplementary Figure S1. Disease rating scale.

Supplementary Figure S2. Suppression of root colonizing *Pythium aphanidermatum* mycelia by GIC41 in hydroponically grown tomato seedlings.

Supplementary Figure S3. Effect of GIC41 on hyphal growth of *Pythium aphanidermatum*.
