# Supplementary material for: Dual benefits of Lysinibacillus xylanilyticus strain GIC41 in mitigating Pythium root rot and enhancing plant growth across cultivation systems
Source: Plant Biotechnol (Tokyo). 2025 Sep 25;42(3):317–26. doi: 10.5511/plantbiotechnology.25.0316a (PMC12573614; doi:10.5511/plantbiotechnology.25.0316a)
Supplement: Supplementary Data [file plantbiotechnology-42-3-25.0316a_s001.pdf]

Supplementary Table S1. Primer and probe sets used for DNA amplification of *Pythium aphanidermatum* and tomato

Name	Sequence	References
Lat52 forward primer	AGACCACGAGAACGATATTTGC	Le Floch et al. 2007
Lat52 reverse primer	TTCTTGCCTTTTCATATCCAGACA	
Lat52 probe	FAM – CTCTTTGCAGTCCTCCCTTGGGCT – BHQ1	
AsPyF	CTGTTCTTTCCTTGAGGTG	Li et al. 2014
AsAPH2B	GCGCGTTGTTCACAATAAATTGC	
kk_aph-Pr2	FAM – CATTTGCCCAGACCATTGCCTC – BHQ1	

References

Le Floch G, Tambong J, Vallance J, Tirilly Y, Lévesque A, Rey P (2007) Rhizosphere persistence of three *Pythium oligandrum* strains in tomato soilless culture assessed by DNA macroarray and real-time PCR, *FEMS Microbiol Ecol*, 61: 317–326

Li M, Ishiguro Y, Otsubo K, Suzuki H, Tsuji T, Miyake N, Nagai H, Suga H, Kageyama K (2014) Monitoring by real-time PCR of three water-borne zoosporic *Pythium* species in potted flower and tomato greenhouses under hydroponic culture systems. *Eur J Plant Pathol* 140: 229–242

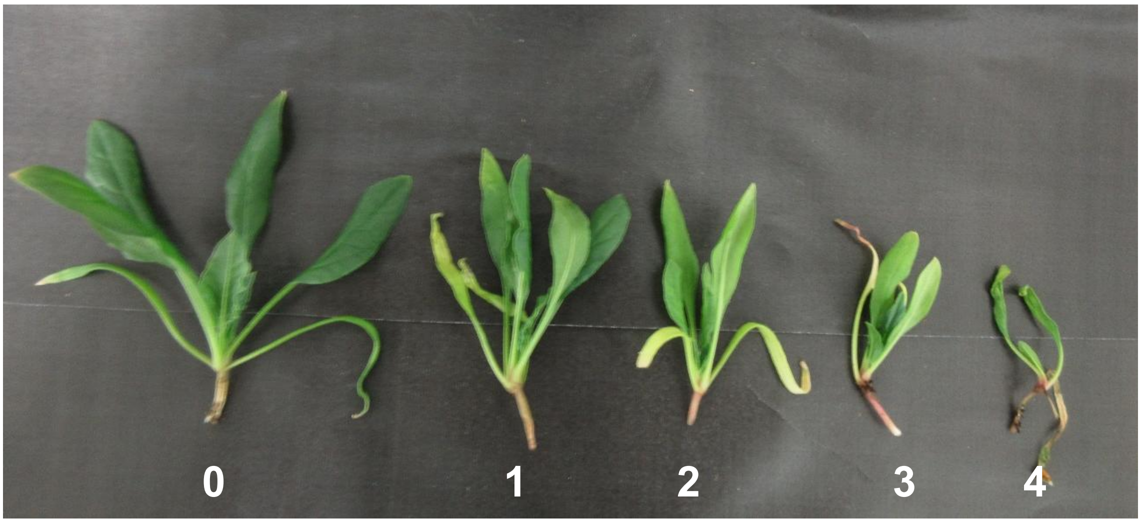

Supplementary Figure S1. Disease rating scale from 0 to 4: 0 = no disease symptoms (healthy), 1 = slightly stunted growth compared to healthy seedlings, 2 = moderate stunting or chlorosis compared to healthy seedlings, 3 = severe stunting or wilting compared to healthy seedlings, 4= completely wilted or dead.

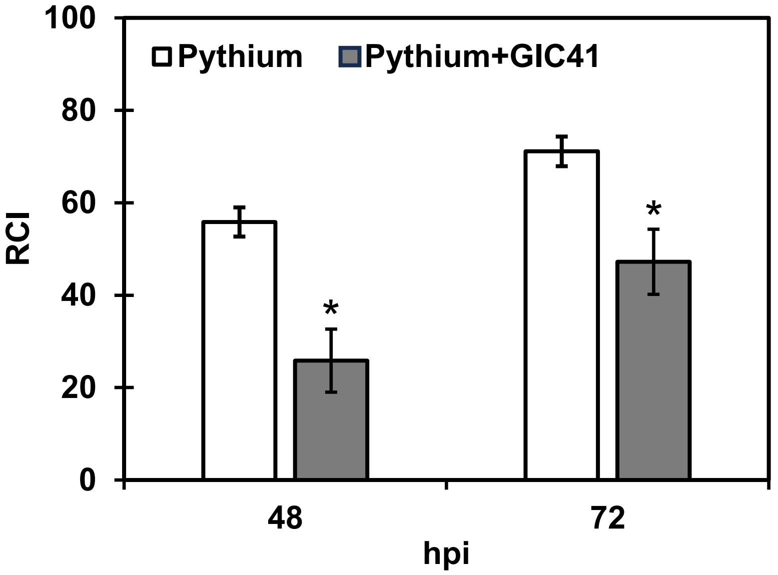

Supplementary Figure S2. Suppression of root-colonizing *Pythium aphanidermatum* mycelia by GIC41 in hydroponically grown tomato seedlings. Error bars represent the standard error from three independent experiments. An asterisk (*) indicates a significant difference between the control (*Pythium*) and GIC41 (*Pythium* + GIC41) treatments according to Student's *t*-test ($p < 0.05$).

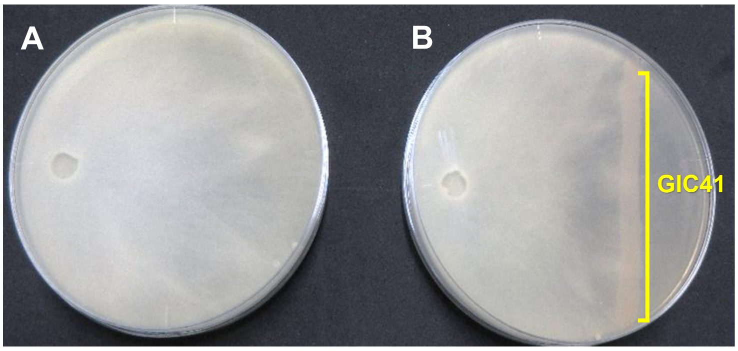

Supplementary Figure S3. Effect of GIC41 on the hyphal growth of *Pythium aphanidermatum*. (A) The culture plate inoculated with *P. aphanidermatum* alone, (B) the culture plate inoculated with *P. aphanidermatum* and GIC41. Hyphae of *P. aphanidermatum* reached the opposite side of the plate in both control and GIC41 treatments.
